# The Classification of Causes of Historical Mortality (CCHM): A proposal of the study of death records

**DOI:** 10.1371/journal.pone.0231311

**Published:** 2020-04-14

**Authors:** Ana-María Sáinz-Otero, Antonio-Jesús Marín-Paz, José Almenara-Barrios

**Affiliations:** 1 Nursing and Physiotherapy Department, Faculty of Nursing and Physiotherapy, University of Cadiz, Cadiz, Spain; 2 Nursing and Physiotherapy Department, Faculty of Nursing, University of Cadiz, Algeciras, Spain; 3 Biomedicine, Biotechnology and Public Health Department, Faculty of Nursing and Physiotherapy, University of Cadiz, Cadiz, Spain; Leibniz Institute for Prevention Research and Epidemiology BIPS, GERMANY

## Abstract

**Objective:**

To compare and contrast the Classification of Causes of Historical Mortality (CCHM) with the International Classification of Diseases 4^th^ Revision (ICD-4) as methodological elements that can be implemented in historical mortality studies.

**Materials and methods:**

We conducted a longitudinal descriptive study of the causes of death in two different localities in Spain, namely, *Cadiz* and *Vejer de la Frontera* (1900–1950), to compare the International Classification of Diseases 4^th^ Revision (ICD-4) and the Classification of Causes of Historical Mortality (CCHM). This study proposes the CCHM and its use in statistical analyses of mortality, especially from the mid-19^th^ century to the second half of 20^th^ century. It is a methodological instrument based on the theoretical precepts of Thomas McKeown, expanded through knowledge gained in studies of historical mortality and contrasted with editions of the ICD.

**Results:**

The results showed several differences between the ICD-4 and the CCHM. The ten main causes of death (CoDs) in the CCHM account for 74.3% in *Cadiz*, compared to 56.6% accounted for by the ICD-4. According to the ICD-4, the number of infectious CoDs exceed the number of noninfectious ones in *Cadiz* every year. On the other hand, based on the CCHM, we observed that while infectious CoD causes of death predominated over noninfectious ones, there was a change in trend, with noninfectious CoDs predominating the following year. During the interval from 1915 to 1937 in *Vejer de la Frontera*, there were 12 deaths due to ill-specified causes (ICD-4: 18.200) and 0 due to ill-defined causes (CCHM: 3.0.0.0).

**Conclusions:**

The CCHM accurately determines the differences between infectious and noninfectious causes of death and explains sociodemographic and health-related aspects in the population and its use in employment, illegitimacy or place-of-death studies. Moreover, it has more advantages, such as the incorporation of new diagnostic expressions, and it can be constantly updated, thus facilitating its use over long periods of time.

## Introduction

Demographic phenomena have been partially linked to humanity and its evolution over time [1]. The availability of natural resources has influenced the growth of the population [2], especially in relation to feeding, as one of the most important restraining factors. In fact, the availability of natural resources remained constant in countries until the beginning of the Industrial Revolution and the development of modern agriculture [3].

In contrast, environmental causes are not the only factors that have modified health, illness, and the consistency of mortality in the population over time. Despite the complexity of the identification of the origin of diseases, Thomas McKeown (1912–1988) established poverty as one of their determining factors, proposing that death at an early age was strongly linked to social conditions of life [1].

The health concept connects the origins of humanity to a comprehension of the factors that have been determined to cause ailments. Social determinants of health have always been adapted to the circumstances of and to the knowledge available in every historical era. Thus, the evolution of the prevailing medical theories have been used to develop a health legislative framework. The discovery of microorganisms as infectious pathogenic entities was supposedly the beginning of the etiological explanation of diseases that affected humanity. Subsequently, medicine initiated the first measures based on the scientific knowledge available to eradicate these diseases. The new and numerous vaccines contrasted with the limited economic resources available and the sanitary infrastructure necessary to fight against the germs. On the other hand, the population did not care about sanitary matters and its preventive aspects.

With the implementation of microbial theory at the end of the 19^th^ century and the application of statistical principles [4], a new ideological current began that was based on hygiene [5]. Health professionals wanted to increase public awareness of hygiene, from the hygiene of the rooms in individuals’ own homes to the sanitation of public roads [6]. In fact, the aim was to prevent the high incidence of infectious diseases and to improve the health knowledge of the population. Health professionals initiated a medicine of strong social character, the progress of which would also depend on the involvement of the public. Thus, “social issues” would soon be a problem of great importance in health disciplines [7, 8].

Then, health professionals’ studies in the population focused on explaining ecological and social models, the risks and protective factors of which modulated the balance. Thus, the findings of these studies reaffirmed the multicausal theory of disease, and variables related to health promotion and the prevention of disease acquired greater importance. To a point, the main theory consists of the following four determinants related to the natural development of the diseases and their consequences in the mortality of the population: the environment, lifestyle and health system as exogenous causes and human biology as an endogenous cause [9]. Currently, the aforementioned model has complemented other theories and shows that studies tend to consider the individual a social entity, such as in the social-ecological model [10].

Mortality and diseases obey numerous biological, political, social, economic, environmental, and cultural factors. They are grouped into exogenous and endogenous conditions, and they should be observed in detail to determine the role of this demographic variable in the study of the population and its consequences [11–15].

### Background of the classifications of the causes of death

Coding diagnostic expressions of *causes of death* (CoDs) according to a standardized nomenclature presents major challenges [16, 17]. In most cases, the person in charge of recording the CoD in the death registry is not a health staff member and is instead integrated into a certain social context [18]; in fact, this person uses expressions from popular medical knowledge [19]. Another problem has been “diagnostic fashion”, when ailments are reported in a particular social determinants of health context, especially in times of epidemic diseases [20]. This approach leads to a lack of precision when classifying diagnostic expressions that are influenced by aspects such as the dichotomy of criteria, a lack of consensus to establish a single classification and the existence of multiple classifications (mainly in the nineteenth century). Therefore, it is difficult to extend an analysis over time of a historical series data as a consequence of modifications of the criteria in the International Classification of Diseases (ICD) and the use of other classifications that have been used over time. One of the classifications that should be highlighted was the one established by Mouriquand in 1930 for infant mortality. He classified causes of death or “dangers” into three large groups: food, infectious, and congenital facts. This classification was widely used by Marcelino Pascua-Martínez and other authors. In particular, the ICD-4 (1929) was composed of 200 rubrics divided into the following 18 groups (Table 1).

**Table 1:** ICD-4.

**1.**	Infectious and parasitic diseases
**2.**	Cancer and other tumors
**3.**	Rheumatism, diseases of nutrition and of endocrine glands, and other general diseases
**4**	Diseases of the blood and blood-forming organs
**5**	Chronic poisoning
**6**	Diseases of the nervous system and sense organs
**7.**	Diseases of the circulatory system
**8.**	Diseases of the respiratory system
**9.**	Diseases of the digestive system
**10.**	Diseases of the genitourinary system and genital tract
**11.**	Pregnancy, birth and puerperal diseases
**12.**	Diseases of the skin and annexes
**13.**	Diseases of the locomotor organs
**14.**	Malformations
**15.**	Diseases of early infancy
**16.**	Diseases of old age
**17.**	Effects of external causes
**18.**	Ill-specified diseases

Source: International Commission for the Decennial Revision of International Nomenclatures of Diseases, 1930.

Thomas McKeown proposed in his book *The Modern Rise of Population* (1976) the first classification, which, according to the epidemiological transition, was based on a separation into two large groups depending on the action or lack thereof of pathogenic microorganisms [1]. From his point of view, given the division that arises from the transmission of these microorganisms via air, water, food and other sources, it is essential to understand the reasons for the decline in mortality since the late 19^th^ century.

In Spain, Bernabeu-Mestre et al. Developed a classification in 2003 that constituted the Modified Causes of Death Classification (CMM). The authors unified it with the ICD-2, taking into account that the study only covered the population between zero and six years old. They established a division based on transmission criteria with up to 3 levels of disaggregation, thus maintaining some harmony with the anatomical criteria of the ICD, as well as the aggregation of a third group based on poorly defined meanings [21].

Pérez-Moreda et al. established another classification as a consequence of the need to combine different CoDs. It consisted of eleven categories. Four CoDs had infectious origins, five had noninfectious origins, one had “ill-defined causes” and the final one was categorized as “other causes” [22].

Therefore, the objectives of our study are to compare and contrast the Classification of Causes of Historical Mortality (CCHM) and the International Classification of Diseases, Fourth Revision (ICD-4) as methodological elements that can be implemented in these types of mortality studies, based on their inclusion of the main epidemiological determinants reflecting the social determinants of health of a population and the evaluation of their use in the statistical analysis of CoDs.

## Methods

This is a longitudinal descriptive study of causes of death in two different localities, namely, *Cadiz* and *Vejer de la Frontera* (southwestern Spain), through the use of the ICD-4 and the CCHM.

### Data source

Data were obtained from all death certificates of the Civil Registries of *Cadiz* (1923–1939) and *Vejer de la Frontera* (1900–1950), with a total of 31,971 and 12,523 deaths, respectively. To establish codes in both classifications, age, sex, cause of death, date in years and place of death were collected in a data frame.

The transcription process is rigorous, as it requires the need to always maintain the exact meaning that appears in the death record, including grammatical and orthographic errors, as well as the aim of facilitating the interpretation of the transcripts in successive historical mortality studies (it is advised to avoid transcribing orthographic signs that appear in the diagnostic expressions to avoid unnecessary duplications). Fortunately, we could recognize most of the diagnostic meanings by consulting several old and current editions of medical dictionaries, which reduced the difficulty involved in cataloging them. Nonetheless, assigning codes involves taking some risks in terms of the nature of the disease, but the use of these dictionaries partially solves the problem.

All data were analyzed with SPSS Statistics^®^ version 24.0 for Windows (IBM Corporation, NY, USA). The distribution and quantification of frequencies have been used through the aforementioned variables by year and by complete period, in addition to their graphical presentation.

### Classification of Causes of Historical Mortality (CCHM)

Although the CMM offers large advantages, it still maintains a certain level of dependence with respect to the ICD. Furthermore, there is no separation between the transmission criteria in the period of pregnancy, birth, and postpartum. Thus, it is appropriate to develop a new classification that addresses the aforementioned issues and is simultaneously an evolution of the CMM with greater independence from the ICD. In this case, we use the ICD-4 because of the convenience of choosing an official classification of causes of death that was equivalent across most of the epidemiological transitions of most developed nations, especially North America and Europe.

Given these factors, we consider feasible the use of the Classification of Causes of Historical Mortality (CCHM) for the quantitative and qualitative study of historical events from the last quarter of the 19^th^ century to the second half of the 20^th^ century. It has proceeded to expand by disaggregating some categories to facilitate a greater capacity for analysis and to allow for comparisons to be made with respect to other classifications of CoD. In the same way, the use of this new classification makes it possible to establish epidemiological and socio-sanitary differences between cities of different geographical locations, e.g., rural or urban town, coastal or inland town, among other separations (according to the researcher). The CCHM can also be used for studies of mortality related to profession, legitimacy and place of death.

Due to the universal nature of the diseases, the CCHM covers an international scope because it is not closed in terms of rubrics. Moreover, it has the advantage of allowing researchers to amplify it with new diagnostic meanings of CoDs that appear in historical series, thus facilitating future revisions of the classification. It is formed by four large groups and four levels of disaggregation. It has infectious and noninfectious disease sections (maintaining McKeown’s postulates) and two more sections: CoDs that do not appear in death certificates and those that are poorly defined [1].

In the case of the infectious disease section, the subgroup of “infectious diseases transmitted by water and food” are divided into typhoid fever and diarrhea and enteritis (with two disaggregations, namely, cholera and dysentery, as a consequence of their epidemic nature). In terms of “airborne infectious diseases”, there are two divisions: “typical of childhood” (characterized by ailments that used to cause epidemic outbreaks in the population) and “respiratory infectious diseases”. In this last group, pneumonia is included, but tuberculosis and influenza have their own codes due to their social determinants of health importance.

Nevertheless, unlike McKeown’s classification, the CCHM adds a category of “vector-borne infectious diseases” because of their morbidity and mortality in countries with more favorable climates for agents that carry pathogens (tropical diseases). The subgroup of “other infections” represents a heterogeneous group, largely classified by etiological and anatomical criteria. Meningitis and poliomyelitis are disaggregated from the group of “other nervous system infections” because of their relationship with the mortality statistics. The “dentition” category remains in the CCHM due to a disuse diagnosis in the late 19^th^ and early 20^th^ centuries that included numerous pathologies in children’s mouths.

The section of noninfectious diseases also presents a disaggregation. It is classified by etiological and anatomical criteria, highlighting the section of “deficiency diseases or nutritional problems”, which includes, separately, marasmus and rickets. “External origin” is another category of special relevance, which is divided into accidental or intentional causes. Moreover, the category of “senility and old age” includes diagnostic meanings related to these terms, without indicating other more precise CoDs (Table 2).

**Table 2:** Classification of Causes of Historical Mortality (CCHM).

**0.**	Without cause of death
**1.**	Infectious diseases
**1.1.**	Infectious transmitted by water and food
**1.1.1.**	Typhoid fever
**1.1.2.**	Diarrhea and enteritis
**1.1.2.1.**	Cholera
**1.1.2.2.**	Dysentery
**1.2.**	Infectious transmitted by air
**1.2.1.**	Own childhood
**1.2.1.1.**	Smallpox
**1.2.1.2.**	Measles
**1.2.1.3.**	Whooping cough
**1.2.1.4.**	Diphtheria
**1.2.2.**	Respiratory system
**1.2.2.1.**	Tuberculosis
**1.2.2.2.**	Influenza
**1.3.**	Infectious transmitted by vectors
**1.3.1.**	Malaria
**1.3.2.**	Exanthematic typhus
**1.4.**	Other infections
**1.4.1.**	Skin and subcutaneous tissue
**1.4.2.**	Nervous system
**1.4.2.1.**	Simple meningitis
**1.4.2.2.**	Poliomyelitis
**1.4.3.**	Organ of the senses
**1.4.4.**	Cardiovascular system
**1.4.5.**	Mouth and its annexes
**1.4.6.**	Urogenital system
**1.4.7.**	Dentition
**1.4.8.**	Respiratory system
**1.4.9.**	Digestive system
**1.4.10.**	Locomotor system
**1.4.11.**	Relating to pregnancy, birth and postpartum
**2.**	Noninfectious diseases
**2.1.**	Deficiency diseases/nutritional problems
**2.1.1.**	Rickets
**2.1.2.**	Marasmus
**2.2.**	Metabolic diseases
**2.3.**	Endocrine diseases
**2.4.**	Cerebrovascular processes
**2.4.1.**	Congestion and cerebral hemorrhage
**2.5.**	Diseases of the nervous system
**2.6.**	Diseases of the cardiovascular system
**2.7.**	Diseases of the respiratory system
**2.8.**	Diseases of digestive system
**2.8.1.**	Stomach
**2.8.2.**	Intestines
**2.8.3.**	Liver and bile ducts
**2.9.**	Diseases of the urogenital system
**2.10.**	Diseases of skin and subcutaneous tissue
**2.11.**	Diseases of locomotor system
**2.12.**	Perinatal pathology (newborn)
**2.12.1.**	Relating to pregnancy, birth and postpartum (mother)
**2.13.**	Congenital defects
**2.14.**	Cancer and tumors
**2.14.1.**	Digestive system
**2.14.2.**	Respiratory system
**2.14.3.**	Female urogenital system
**2.14.4**	Male urogenital system
**2.14.5.**	Skin
**2.14.6.**	Locomotor system
**2.14.7.**	Other organs and unspecified organs
**2.14.8.**	Benign tumors
**2.15.**	External origin
**2.15.1.**	Due to accidental causes (e.g., suffocation, poisoning, burns, polytraumatism; in other words, they do not know if the events were causal, provoked or violent)
**2.15.2.**	Traumatisms, weapon wounds and suicides. Intentional or provoked
**2.16.**	Senility and old age
**3.**	Ill-defined diseases

Source: Authors’ elaboration, 2019.

In a mortality database, to assign a CCHM code for the different diagnostic expressions that appear in the death certificates, it is important to make an automatic assignment (particularly when diagnostic expressions have been coded with this classification in other previous studies). If they are not coincidental, we follow two criteria exposed by Bernabeu-Mestre et al. [21]: “if the diagnostic expression refers to an anatomical criterion, it will be assigned to the *Other diseases of the X system* category, maintaining its maximum level of disaggregation” and “if the CoD combined several symptoms, etiologies, or location, the previous criterion will be followed if it is not possible to make an automatic classification”. We need to follow with another criterion: “If in the CoD section of the death record has no information, this lack of this information is automatically coded as 0.0 (ICD) or 0.0.0.0 (CMM)”. Furthermore, the classification criteria of the ICD-6 [23] will be followed rigorously and in order, in case of the appearance of two or more diagnostic expressions in a single cause of death (Table 3).

**Table 3:** Classification criteria of the ICD-6.

**1.**	If a pathological condition was often an immediate complication of another, then the primary disease would be preferred to the complication
**2.**	If the abovementioned scenario were not the situation and one of the pathological conditions mentioned was an accident, poisoning, or other act of violence, then preference should be given to these expressions
**3.**	If none of these situations existed but there was a significant indication of a difference in the apparent severity of the pathological conditions, the most serious entity would be preferred
**4.**	If none of the situations mentioned above occurred but one of the causes adduced is an infectious or parasitic disease, preference should be given to it
**5.**	If they are chronic diseases and none of the previous rules is adjusted, but there is an indication in the certificate about the duration of the causes, then the disease with the longest duration should be preferred
**6.**	Finally, if none of the situations described above were applicable, the preference should be attributed to the pathological condition mentioned first

Source: WHO, 1948.

### Ethics

When information is collected from death records between 1900–1950, approval by an ethics committee is not necessary. Spanish legislation allows for the documentary observation of archives and the collection of confidential data of people who died at least 25 years ago [24]. Given the legislation, there is no legal requirement for anonymity of records, nevertheless no identifying information of deceased persons was entered into the dataset.

## Results

The study of deaths in the Spanish coastal city of *Cadiz* (1923–1939) and the inland town of *Vejer de la Frontera* (1900–1950) shows several differences between the CCHM and the ICD-4. First, the main cause of death in *Cadiz* was “other heart diseases” (ICD-4) and “diseases of the cardiovascular system” (CCHM). Nonetheless, there are differences in their percentages (11.5% versus 17.7%), due to the division of cardiac causes in the ICD-4. The same case occurs between deaths from “bronchopneumonia, including capillary bronchitis” (ICD-4) and “infectious diseases of respiratory system” (CCHM) or between “diarrhea and enteritis (under two years of age)” (ICD-4) and “diarrhea and enteritis” (CCHM). Therefore, it is observed that the ten main CoDs in the CCHM account for 74.3% of the CoDs in *Cadiz*, compared to 56.6% accounted for by the ICD-4 (Table 4).

**Table 4:** Main CoDs according to the ICD-4 and the CCHM. Cadiz (1923–1939).

ICD-4		CCHM	
Cause of Death	Percentage	Cause of Death	Percentage
Other heart diseases (code 7.95)	11.5%	Diseases of cardiovascular system (code 2.6.0.0)	17.7%
Respiratory tuberculosis (code 1.23)	10.3%	Tuberculosis (code 1.2.2.1)	13.2%
Cerebral hemorrhage, embolism or cerebral thrombosis (code 6.82)	7.8%	Infectious diseases of respiratory system (code 1.2.2.0)	10.5%
Bronchopneumonia, including capillary bronchitis (code 8.107)	6.7%	Other infectious of cardiovascular system (code 1.4.4.0)	6.6%
Myocardial diseases (code 7.93)	4.9%	Congestion and cerebral hemorrhage (code 2.4.1.0)	6.1%
Simple meningitis (code 6.79)	3.8%	Diarrhea and enteritis (code 1.1.2.0)	5.1%
Diarrhea and enteritis (under two years old) (code 9.119)	3.4%	Other infectious of nervous system (code 1.4.2.0)	4.7%
Other diseases peculiar to early childhood (code 15.161)	3.0%	Marasmus (code 2.1.2.0)	4.0%
Ill-specified diseases (code 18.200)	2.7%	Other infectious of urogenital system (code 1.4.6.0)	4.0%
Arteriosclerosis, except diseases of coronary arteries (code 7.97)	2.5%	Senility and old age (code 2.16.0.0)	2.4%
Total	56.6%	Total	74.3%

Source: Civil Register of Cadiz. Authors’ elaboration, 2019.

On the other hand, the main cause of death in *Vejer de la Frontera* was diarrhea and enteritis in both classifications, but the percentage is lower in the ICD-4 due to the fact that it only covers deaths under the age of two. Nevertheless, the percentages for the CoDs "infectious diseases of the respiratory system" and "diseases of cardiovascular system" (CCHM) are approximately twice as high as those for "bronchopneumonia, including capillary bronchitis" and "other heart diseases" (ICD-4), respectively. In this case, similar to *Cadiz*, a greater percentage of the ten main CoDs are included in the CCHM than in the ICD-4 (Table 5).

**Table 5:** Main CoDs according to the ICD-4 and the CCHM. Vejer de la Frontera (1900–1950).

ICD-4		CCHM	
CoD	Percentage	CoD	Percentage
Diarrhea and enteritis (under two years old) (code 9.119)	10.2%	Diarrhea and enteritis (code 1.1.2.0)	13.2%
Bronchopneumonia, including capillary bronchitis (code 8.107)	8.0%	Infectious diseases of respiratory system (code 1.2.2.0)	13.1%
Respiratory tuberculosis (code 1.23)	6.5%	Tuberculosis (code 1.2.2.1)	9.3%
Cerebral hemorrhage, embolism or cerebral thrombosis (code 6.82)	6.2%	Diseases of cardiovascular system (code 2.6.0.0)	8.7%
Influenza (code 1.11)	4.0%	Congestion and cerebral hemorrhage (code 2.4.1.0)	5.0%
Other heart diseases (code 7.95)	3.8%	Influenza (code 1.2.2.2)	4.0%
Other diseases peculiar to early childhood (code 15.161)	3.3%	Perinatal pathology (newborn) (code 2.12.0.0)	3.2%
Congenital weakness (code 15.158)	3.2%	Rickets (code 2.1.1.0)	3.1%
Rickets (code 3.63)	3.1%	Marasmus (code 2.1.2.0)	3.1%
Diarrhea and enteritis (two years old or more) (code 9.120)	3.1%	Simple meningitis (code 1.4.2.1)	3.0%
Total	51.4%	Total	65.7%

Source: Civil Register of Vejer de la Frontera. Authors’ elaboration, 2019.

**Fig 1:**
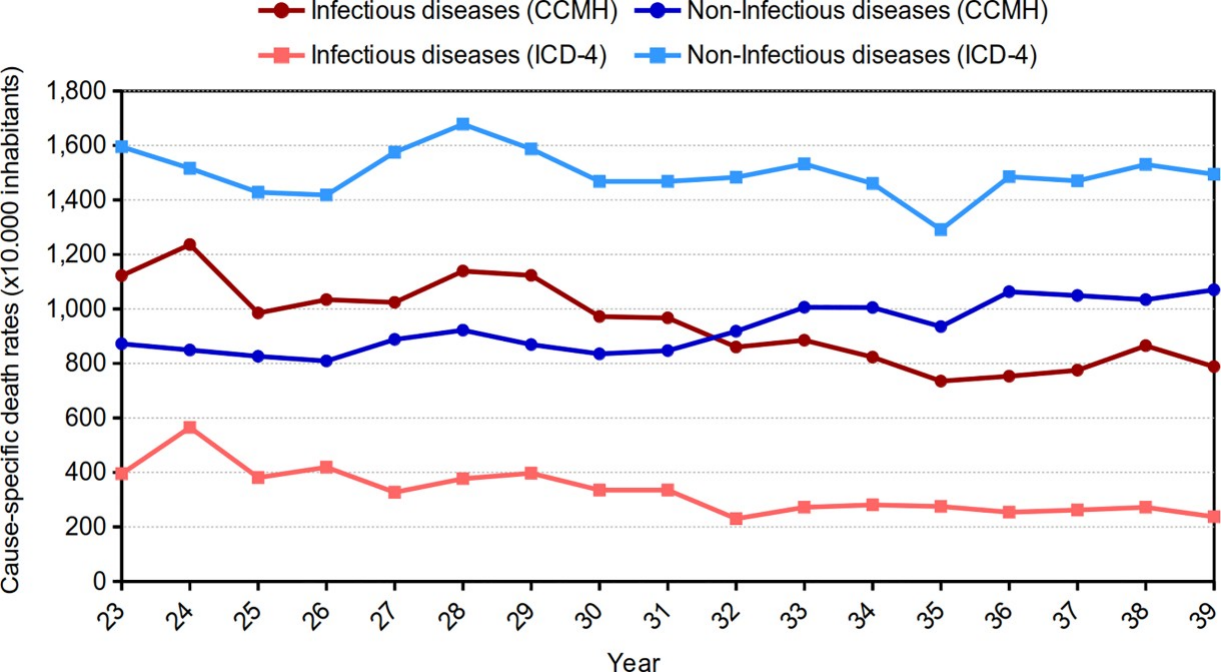
Evolution of cause-specific death rates in infectious (first group) and noninfectious (other groups) diseases per year by the ICD-4. Cadiz (1900–1950). Source: Civil Register of Cadiz. Authors’ elaboration, 2019.

Second, the epidemiological transition observed differs depending on the classification. Through the ICD-4, it is observed that the number of infectious CoDs exceeds the number of noninfectious ones in Cadiz every year. In fact, there is a difference of 1,000–1,200 deaths per ten thousand inhabitants between the two groups during the whole period in this case. On the other hand, a difference trend is observed when the CCHM is used. Until 1931, infectious CoDs predominated over noninfectious ones. However, there is a change in trend, and the following year, noninfectious CoDs predominate (Fig 1).

**Fig 2:**
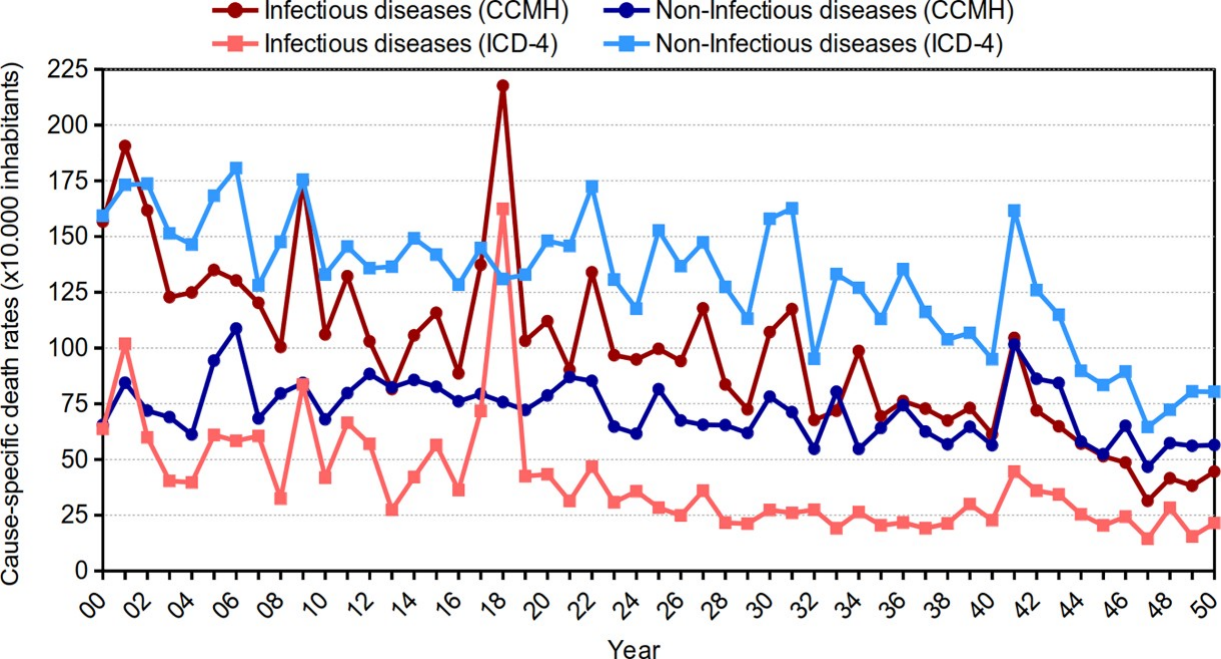
Evolution of cause-specific death rates in infectious and noninfectious diseases per year by the CCHM. Vejer de la Frontera (1900–1950). Source: Civil Register of Vejer de la Frontera. Authors’ elaboration, 2019.

The annual evolution of infectious diseases in both classifications is similar in *Vejer de la Frontera*. Nonetheless, cause-specific death rates are lower in the ICD-4 with respect to the CCHM. There is also a completely different trend for noninfectious diseases. High rates are recorded in the ICD, surpassing those of infectious diseases every year (except 1918). On the other hand, the annual rates of noninfectious diseases, according to the CCHM, remain below those of infectious diseases for most of the period analyzed until 1941, when the trend is reversed (Fig 2).

**Fig 3:**
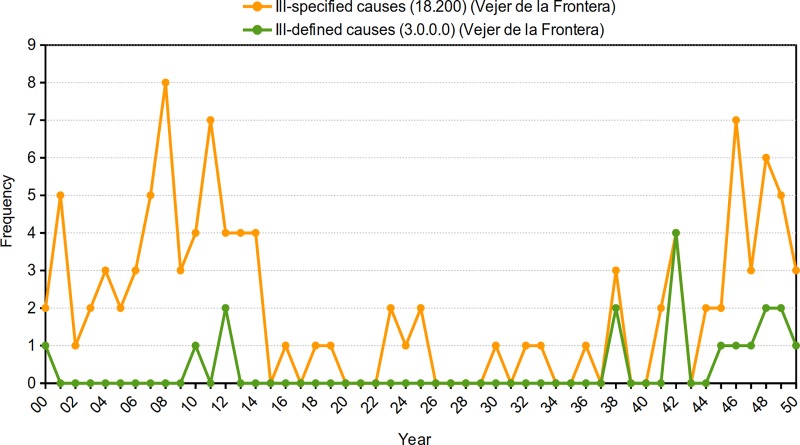
Annual distribution of all deaths due to ill-specified causes according to the ICD-4 and for ill-defined causes according to the CCHM. Vejer de la Frontera (1900–1950). Source: Civil Register of Vejer de la Frontera. Authors’ elaboration, 2019.

Finally, during the interval from 1915 to 1937 in *Vejer de la Frontera*, there were 12 deaths due to ill-specified causes (ICD-4: 18.200) and 0 due to ill-defined causes (CCHM: 3.0.0.0). The annual frequency for ill-specified causes (ICD-4: 18.200) increased considerably at extreme dates as a consequence of the specificity that the different editions of the ICD were acquiring (in the first years of the 20th century) and its subsequently outdated previous editions (1940s). This disadvantage is not seen in the case of the use of the CCHM: Ill-defined causes never surpassed ill-specified causes in the years studied. An examination of all deaths from these CoDs indicated that the number of deaths registered in this locality between 1900–1950 were 18 (CCHM) and 106 (ICD-4) (Fig 3).

**Fig 4:**
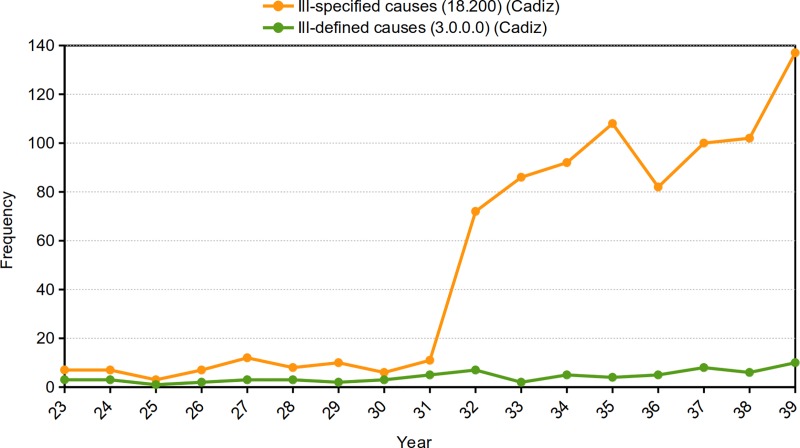
Annual distribution of all deaths due to ill-specified causes according to the ICD-4 and for ill-defined causes according to the CCHM. Cadiz (1923–1939). Source: Civil Register of Cadiz. Authors’ elaboration, 2019.

There was a greater difference in *Cadiz* between both codes, as the ICD-4 was outdated over time. Ill-specified diseases were the ninth-leading cause of death. Despite maintaining similar values in the first years, beginning in 1932, the ill-specified diseases of the ICD-4 began to increase in an important way. Specifically, the largest difference occurred in the final year, in which 137 deaths were coded as ill-specified causes and only 10 deaths were coded as ill-defined causes (Fig 4).

## Discussion

Researchers have created several methods to code CoDs due to the variability in terms of diagnostic expressions. In addition, they have wanted to establish ways relating statistical studies to the historical context of the population [25].

Thomas Sydenham was the first person to typify CoDs in the 17th century [26]. In the First International Congress of Statistics (1853), William Farr created a classification of CoDs of a universal character and divided it into five chapters [27]. Unfortunately, it was not applied in many countries [28]. He was not the only person who presented a nomenclature project: Marc d'Espine proposed another classification very similar to that of the previous author in the same congress, dividing CoDs according to their features. Nonetheless, Jacques Bertillon created the definitive classification, keeping the anatomical criteria of the CoDs presented by William Farr [29]. The ICD became a fundamental instrument for CoDs, as it was accepted and used throughout North America, partially in South America, and in Europe since 1897 [30].

After its implementation, an international commission began to review it in 1900. They established a 2^nd^ revision nine years later. They approved the application of changes with a decennial periodicity to allow for the statistical treatment of the data. Another important aspect was the increase in the number of representatives from different countries to establish a consensus for each of the rubrics of the classification. Furthermore, they implemented more CoDs pertaining to infectious diseases, as well as the differentiation of terms in relation to the age of the deceased, especially in cases of infant and juvenile mortality [31].

In 1929, the International Commission created a specific classification of causes of stillbirth in the 4^th^ revision. In addition, they prepared a series of recommendations for the following sessions. In each of them, they highlighted the reorganization of the ages of death for a better comparison of statistical results, the standardization of death certificates and the training of medical students for the proper processing of this kind of certificate [32]. The successive revisions of the ICD improved the statistical techniques of data collection regarding the CoDs, but the main problems were not solved [33–34].

Nevertheless, the ICD has some disadvantages regarding the terminology of the causes of mortality and their coding. Many researchers developed classifications of diseases adapted to the work they were doing [35], even taking into account that the possibilities of bias derived from the classification itself will always be present [36].

The International Conference of the History of the Causes of Death Registry, which took place in Bloomington in November 1993, or the publication of a monograph issue of Diagnostic Expressions and Causes of Death by the Association of Historical Demography (1993) show the wide range of disciplines involved in historical studies on death registers. For that reason, there are several arguments that explain the interest in CoDs among studies throughout history: from the explanation of the demographic evolution of a population to the modification of health strategies based on this knowledge of CoDs [37].

The distribution and behavior of the CoDs throughout the period studied is not homogeneous and presented variations in their frequencies. On the one hand, there was a change in the leadership of the causes of death, from a pattern of high mortality due to infectious diseases to the predominance of chronic or degenerative diseases. On the other hand, mortality crises are discovered that, in some cases, do not correspond to those that have occurred in other cities of the country or even in other countries. It is also mediated by age: the prevalence of one disease over another could be related to age at the time of death, depending on whether the person is under one year of age or is at least a year old.

The nomenclature attributed to the CoDs has evolved over the years from nonspecific terms or with another denomination to more precise or current diagnostic meanings. Likewise, depending on the sanitary and cultural conditions, the registration of infectious diseases such as *tuberculosis*, *pneumonia* or *typhoid fever* is frequent at the beginning of the 20^th^ century, as well as various expressions attributed to death that are no longer used (*gastric fever*, *cerebral softening* or *intestinal cold*). Other nonspecific terms (*infectious fever* and *pyrexia*) also appeared in death registers or CoDs related to old age (for instance, *senile atrophy*, *senile weakness* and *senile consumption*).

With improvements in the diagnosis, prevention and treatment of diseases, some diseases disappeared from death records. These absences were accompanied by an increase in the specificity of the CoDs, attributed mainly to the affected anatomical region, such as *malaria pernicious fever and cerebral form*. In the same way, other death certificates have unconventional CoDs for war reasons. Close to the 1940s, new meanings, as well as more modern diagnostic classifications, appeared: *Vitamin deficiency*, *hematemesis* or *anaphylactic shock*. Some expressions replaced others in decline (for instance, *bronchiolitis* instead of *capillary bronchitis*), and the intermediate-CoD was progressively added.

The impact of the Spanish flu pandemic of 1918–1919 was one of the main methodological problems due to its forms of registration and consequences for the population, including children [38], due to a lack of reliability in the annotations of CoDs in death records. Influenza was related to other causes linked to complications of the disease (bronchitis, bronchopneumonia and pneumonia) [1]. In fact, according to Álvarez Pardo et al., Erkoreka, Hernández-Ferrer, Herrera-Rodríguez, and Porras-Gallo [39–45], it was possible to register cases of death attributed to influenza with another CoDs to prevent a state of panic in the general population. Another methodological problem is war as a demographic phenomenon. To demonstrate, the Spanish Civil War (1936–1939) and the Second World War (1939–1945) affected several death records, such as the delay of the inscriptions, CoDs related to pathologies attributable to the war and the alteration of information as a consequence of postwar political repression [46].

To clarify the process of the identification of CoDs and their coding, some of them were studied, and they will be added here as examples, taking as reference the codes corresponding to the ICD (ICD-4) and the CCHM. Sometimes the same expression of CoD is coded differently according to the diagnostic currents of the time, and this change is appreciated in the various reviews of the ICD. This change in meaning implies an ambiguity in the codification in mortality studies depending on the years analyzed (for instance, the *tabes dorsalis*). The use of a specific edition of the ICD depends on the time series in the study; therefore, a transcription bias is inevitable when mortality due to a given cause is compared with that of other studies, which is an aspect that it is corrected through the use of the CCHM.

Additionally, despite being nonspecific as a secondary disease, *cachexia* in the ICD-4 can be specifically coded depending on age: under one year (code 15.158), one year until sixty-nine years (code 18.200) and seventy years and more (code 16.162). The majority of deaths from this cause were observed during the war and postwar period in the CCHM. As a result, it is assumed that they are related to generalized malnutrition and are thus assigned the code 2.1.0.0.

*Marasmus* (ICD-4: 15.161, CCHM: 2.1.2.0) is a nonspecific term that has remained in the past. Like different diseases, both infectious and noninfectious, it is complicated to understand, making it difficult to codify. Because of this, its acceptance varies according to the classification of causes of death used and the socio-economic and health context. In the mid-20th century, there were some medical articles that described it as a deficiency disease, thus reaffirming the repositioning of marasmus in this category of the CCHM [47]. It is considered feasible to include *senile marasmus* in the corresponding categories of the classifications oriented toward old age and senility (ICD-4: 16.162, CCHM: 2.16.0.0) because the meaning is encompassed in a specific age group. Similarly, according to Bernabeu-Mestre et al. [48], *chlorosis* (ICD-4: 4.71, CCHM: 2.1.0.0), similar to *marasmus* and *hysteria*, was a diagnostic expression that had its peak in the 19th century. Its use was reduced according to the improved diagnostic techniques in the 20th century, so it was consigned as a secondary ailment between infection or deficient blood disease.

There are divisions based on the acute or chronic nature of a disease. The same fatal disease has an infectious etiology in some individuals, while in others, it depends on noninfectious factors. An example of this would be *acute nephritis* (ICD-4: 10.130, CCHM: 1.4.6.0) and *chronic nephritis* (ICD-4: 10.131, CCHM: 2.9.0.0). On the other hand, there are divisions according to age: *spasmodic laryngitis* (ICD-4: 8.105, CCHM: 1.2.1.0) is characterized as a disease that affects children under seven years old. Moreover, some diagnostic expressions classified as ill-defined CoDs in the ICD-4 (code 18.200) are assigned to the same category in the CCHM, for example, *dropsy* (code 2.6.0.0).

CoDs and, in a strict sense, the diagnostic expressions that inform us about them, are important elements in the demographic analysis of mortality, despite their problems and limitations [49]. The grouping of the CoDs in the proposed classification does not precisely coincide with that of the others mentioned before, but it is useful for studying epidemiological transitions in many countries. This is especially interesting if the historical versions of the ICD present problems in categorizing infectious and noninfectious diseases by following anatomical and etiological criteria. Similarly, there are statistically important differences between one CoD with respect to another, depending on the classification that may be chosen.

In historical mortality studies, it is possible to use both the CCHM and the ICD, in addition to knowing the differences between the two classifications. They provide us with more information on the causal mechanisms that affect the evolution and peculiar characteristics of mortality. In this vein, ill-defined causes provide special-interest data that help determine the approximate degree of accuracy of a classification of CoDs. The ICD and its respective editions were adapted to the changes of the period in which they were published. Therefore, their degree of accuracy depends on the time in which each edition was valid. In spite of the results presented, it should be noted that there are other two limitations in this current study: the lack of data on several death certificates and the partially very low numbers of codes analyzed.

## Conclusions

The Classification of Causes of Historical Mortality (CCHM) is based on the McKeown thesis and would be an alternative to the International Classification of Diseases (ICD). The use of the ICD does not allow for the study of the epidemiological transition in populations due to its anatomical and etiological criteria. Therefore, the CCHM is a useful methodological instrument for the statistical analysis of causes of death in historical mortality studies, based on the division of diseases according to infectious or noninfectious etiology.

In addition, diagnostic expressions evolved according to medical advances, an aspect contemplated in the CCHM as a dynamic element for studies over long periods of time. In fact, this research showed that coding causes of death by the CCHM enormously decreased the frequency of “ill-defined causes” (code 3.0.0.0) compared to the frequency of “indeterminate causes of death” corresponding to the ICD-4 (code 18.200).

Nonetheless, it is necessary to publish more historical mortality studies to confirm the suitability of the CCHM compared with other versions of the ICD, as well as the unification of criteria to facilitate the understanding of the results between causes of death in different studies. Finally, the proposed classification establishes new perspectives to understand the social determinants of health in a population over a certain period of time.
